# Temporally aligned segmentation and clustering (TASC) framework for behavior time series analysis

**DOI:** 10.1038/s41598-024-63669-6

**Published:** 2024-06-28

**Authors:** Ekaterina Zinkovskaia, Orel Tahary, Yocheved Loewenstern, Noa Benaroya-Milshtein, Izhar Bar-Gad

**Affiliations:** 1 Gonda Multidisciplinary Brain Research Center, Bar-Ilan University, Ramat Gan, Israel; 2 Department of Psychological Medicine, The Neuropsychiatric Tourette Clinic, Schneider Children’s Medical Center of Israel, Petah Tikva, Israel; 3 School of Medicine, Faculty of Medical and Health Sciences, Tel Aviv University, Tel Aviv, Israel

**Keywords:** Animal behaviour, Computational science

## Abstract

Behavior exhibits a complex spatiotemporal structure consisting of discrete sub-behaviors, or motifs. Continuous behavior data requires segmentation and clustering to reveal these embedded motifs. The popularity of automatic behavior quantification is growing, but existing solutions are often tailored to specific needs and are not designed for the time scale and precision required in many experimental and clinical settings. Here we propose a generalized framework with an iterative approach to refine both segmentation and clustering. Temporally aligned segmentation and clustering (TASC) uses temporal linear alignment to compute distances between and align the recurring behavior motifs in a multidimensional time series, enabling precise segmentation and clustering. We introduce an alternating-step process: evaluation of temporal neighbors against current cluster centroids using linear alignment, alternating with selecting the best non-overlapping segments and their subsequent re-clustering. The framework is evaluated on semi-synthetic and real-world experimental and clinical data, demonstrating enhanced segmentation and clustering, offering a better foundation for consequent research. The framework may be used to extend existing tools in the field of behavior research and may be applied to other domains requiring high precision of time series segmentation.

## Introduction

Neuroscience experiments investigating behavior and its underlying mechanisms have been traditionally confined to observing and quantifying either constrained movement or limited subsets of it^1^, both in experimental animal models and in human subjects. Behavior quantification in those scenarios could be approximated either by using the experimental setup, e.g., the timing between a cue and a lever press can help approximate timing and characteristic of a reaching movement^2^, or be meticulously labeled by a trained expert^1,2^. Moreover, it is common to group behaviors over repetitive trials, which allows to investigate behavior and neuronal characteristic of each group^1^. However, such methods cannot be applied to natural-behavior experiments due to the lack of the strict setup and trial organization, nor can the manual labeling scale up to complex behavioral or clinical experiments. Recent advancements in sensor technology, signal analysis, and especially machine learning, have catalyzed a shift towards automated approaches for behavior analysis^3^. Despite recent advances, the field still encounters major challenges, such as the complexity of behavior deconstruction and the high specificity of the existing solutions^4,5^. In this study we aim to provide a generalized tool that can be used broadly for a variety of data and coupled with existing pipelines.

**Figure 1:**
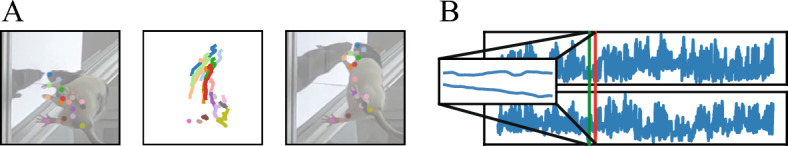
Behavior segmentation. An illustration of a single behavior motif within the context of a continuous sensor (single video camera) recording. (**A**) 17 landmarks’ tracking of a freely behaving rat. Beginning of a movement (left), progression of the tracked landmarks in time (90 frames) (center) and end of the movement (right). (**B**) A zoom out from the motif's segment to the continuous signal of a single landmark’s x (top) & y (bottom) values (green and red lines—beginning and end of the segment respectively).

Behavior quantification forms the basis for analyzing the mechanisms governing normal and pathological behaviors. Two crucial steps towards behavior quantification are segmentation and clustering^5^. Segmentation is a process that entails dividing the data embedded within a continuous time series into short periods (segments), each representing a recurring sub-behavior, often termed behavior motif (Fig. 1). The latter refers to assigning such segments into meaningful clusters, each representing a single underlying sub-behavior. Segmentation and clustering are often approached separately, but when coupled can enhance the results of both processes^6^: clustering can improve segmentation by providing a clear organization of the existing segments within the continuous data, and conversely, precise boundary placement provides well-defined segments for the clustering leading to more meaningful groups^7^.

Behavior data is acquired via a myriad of sensors ranging from video cameras to inertial measurement units and is transformed into a multidimensional time series. This aligns the task of behavior quantification with existing solutions for time series segmentation and clustering^8^. However, the temporal sensitivity and atypical patterns inherent in behavioral data related to experimental and clinical research form specific challenges: firstly, neural changes associated with the behavior manifest on a sub-second scale, and to find neural correlates the behavior segmentation must adhere to this high precision^9^. Secondly, subjects of the research often exhibit abnormal movement profiles, characterized by non-smooth, rapid and unexpected motions^10,11^. Lastly, similarly to how neuronal data can be aligned using experimental parameters during repetitive trials, the profile of the behavior segments must yield parameters that may be applied to the underlying neural activity to align it during multiple executions of non-predefined behavior in the absence of the setup cues^9^.

Behavior motifs can span multiple time scales: in the field of Human Activity Segmentation (HAS), sub-behaviors are typically viewed as a continuous bout of the same activity, such as a period of walking or sitting^12^. In the experimental and clinical domains motifs are typically much shorter with a time scale of a second, involving patterns such as a single complete cycle of walking, or a sub-second time scale, focused on individual sub-movements^13,14^. Behavior is composed of multiple motifs that vary temporally and in complexity both between different motifs, e.g., the mean duration of a grooming stage is shorter than a body turning cycle, and within multiple occurrences of the same motif, e.g., body turning is executed at varying speeds each time. This variability leads to major challenges in both segmentation and clustering.

Our framework draws inspiration from a corpus of prior research in animal and human behavior quantification. Matrix Profile (MP) is an elegant solution for identifying similarity between segments which can form a basis for motif discovery in time series^15,16^. Since its introduction, it has been widely used in a variety of applications including behavior quantification^17^. MP has been used to find k-best motifs^18^ and can be used for time series segmentation^17^. Noticeable progress in animal behavior segmentation and clustering was presented in frameworks such as MotionMapper^19^, B-Soid^14^ and Motion Sequencing (MoSeq)^13^ (including the recent keypoint-MoSeq^20^). These solutions constitute benchmark results but may lack in generalizability as they are often tailored to the needs of the lab developing it. In this study, we extend the capabilities of these frameworks by using temporal alignment. This family of techniques is used for sequence alignment and similarity measurement and allows accommodating sequences of variable durations and execution velocities. Temporal alignment has been widely used in the field of speech recognition^21,22^ and it ranges from linear alignment that optimizes the resampling and shift factors^23^ to the non-linear Dynamic Time Warping (DTW), which, unless regularized, provides the best possible point-wise match for any two sequences^24^. The latter forms a basis for DTW Barycenter Averaging (DBA) algorithm that can be applied to sequences’ clustering^25^. DBA iteratively refines an average sequence to minimize its distance to a cluster of time series. These methods have also been previously shown to aid time series segmentation and clustering in tasks such as HAS^26–28^. In this study, we utilize iterative temporal alignment to automate behavior segmentation and clustering to provide motif boundary refinement robust to temporal variability.

## Results

**Figure 2:**
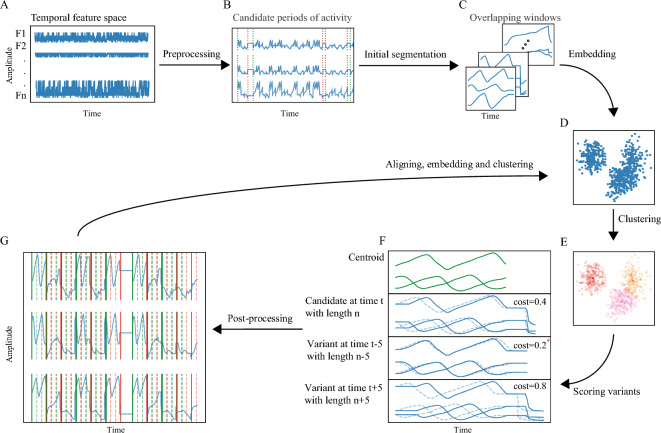
TASC framework flow. (**A**) Multidimensional continuous behavior time series. (**B**) Candidate periods of activity identified following normalization. (**C**) Initial segmentation. (**D**) Embedding in latent space followed by (**E**) clustering. (**F**) For each segment, variants are generated from its temporal neighbors and are scored relative to its cluster’s center using linear alignment. The variant is “shifted” and “warped” such that the distance between the aligned version and the center is minimal, which is equivalent to the maximal coincidence of the sequences. Two example variants are shown, for each one the dashed lines mark the original neighbor, and the solid lines—the aligned version. (**G**) Elimination of overlaps and filling of significant gaps for highly scoring segments. Dashed lines mark overlapping candidate segments, solid—the best choice after overlap elimination. Green and red lines mark the beginning and end of segments respectively.

The TASC framework provides temporal refinement of data segmentation and clustering, with an emphasis on temporal continuity and variability of behavior motifs. The prerequisites for applying TASC are: (1) an embedding method, denoted as *F*_*E,*_ and (2) a cluster assignment method, denoted as *F*_*C*_. As a first step, a dataset of multidimensional time series (Fig. 2A) is normalized, and candidate *activity periods* are identified by applying a cumulative low-pass filter across features and thresholding to prevent overrepresentation of periods of inactivity and their dominance in the resulting clusters (Fig. 2B). An initial set of segments’ boundaries and labels is either provided externally or is set by TASC (Methods—Initial segmentation and clustering). For the latter, an appropriate window size needs to be provided, such that would describe an average timescale of the expected motifs of interest. A segment is considered valid if at least 90% of its duration falls within an activity period (Fig. 2C). The segments are embedded into a low-dimensional space (*F*_*E*_) (Fig. 2D) and clustered (F_C_) (Fig. 2E). Prior to calculating the clusters’ centroids using a method of choice (existing median by default) TASC calculates pairwise distances within clusters to preserve only members within a provided factor ($\gamma$) of standard deviation from the median distance (Methods—Outlier removal) leading to better defined centroids. Additional parameters that require the user’s definition are a range of temporal neighbors’ offsets, M, and lengths, L, which together define the region and duration of the temporal neighbors to consider in the following step. After all the temporal neighbors within the provided range are evaluated against corresponding clusters’ centroids using linear alignment, the highest scoring cluster members (segments) are selected while eliminating any overlaps using the alignment costs converted into scores (Fig. 2F). Significantly large gaps are removed, by identifying the best scoring segments within those gaps and reassigning them to the segment pool following a thresholding relative to the closest cluster (Methods—Gap reassignment) (Fig. 2G). These segments are embedded following resampling of the aligned segments to a uniform length averaged over all segments’ lengths and labeled, and the results serve as the input for the next iteration of the framework. The pseudocode for the TASC main algorithm appears in Box 1, and a full description of TASC algorithms, along with a summary of the parameters, can be found in “Methods” section and Table 1.

**Table 1:** TASC parameters summary.

Parameter	Description
$M=({m}_{\text{min}},{m}_{\text{max}})$	Range of offsets for temporal neighbors
$L= ({l}_{\text{min}},{l}_{\text{max}})$	Range of segments’ length to consider when evaluating temporal neighbors
α	A weight by which to penalize the linear alignment. The bigger the penalty, the less warping is allowed. Can be passed as an array of values different for each epoch
$\gamma$	Factor affecting what is considered an outlier within a cluster. Tied to the standard deviation of the pairwise distances in cluster
Centroid method	Which method to use to calculate centroids
Centroid existing	Whether to use as centroid the calculated average (according to one of the supported methods) or the closest to it member of the cluster

Box 1. TASC Pseudocode **Definitions:**

D-dimensional signal     $$S$$

Initial boundaries     $${T}_{i}=\left({t}_{{B}_{i}}, {t}_{{E}_{i}}\right),1\le \text{i}\le {\text{N}}_{segments}$$

Range of segment offsets     $M=({m}_{\text{min}},{m}_{\text{max}})$

Range of segment lengths     $L= ({l}_{\text{min}},{l}_{\text{max}})$

Penalty weight for LinearAlign     $\alpha$

Filter parameter for clusters     $\gamma$

**Algorithm:**

1. $${F}_{E}\left(S\left[{T}_{i}\right]\right)\to {E}_{i}$$, $${F}_{c}\left({E}_{i}\right)\to {C}_{k},1\le k\le {N}_{clusters}$$

2. *Outlier removal* using $\gamma$

3. Calculate *centroids* for the filtered clusters

4. Generate all motif variations:

   For each *i*, $$m,l\in {\text{N}}_{segments},M,L$$:

      $${v}_{i,m,l}=\left({t}_{{B}_{i}}+m, {t}_{{E}_{i}}+m+l\right)$$

$$5. {Scores}_{i,m,l}, {Params}_{i,m,l} \leftarrow LinearAlign\left({v}_{i,m,l},centroid\left({C}_{k}\right),\alpha \right)$$

6. Dynamically maximize total score while eliminating overlaps.

7. Align the selected segments using the parameters, re-embed and re-cluster.

8. Fill in gaps according to the conditions.

9. Unless convergence is achieved, reiterate.

### TASC assessment using a semi-synthetic dataset

**Figure 3:**
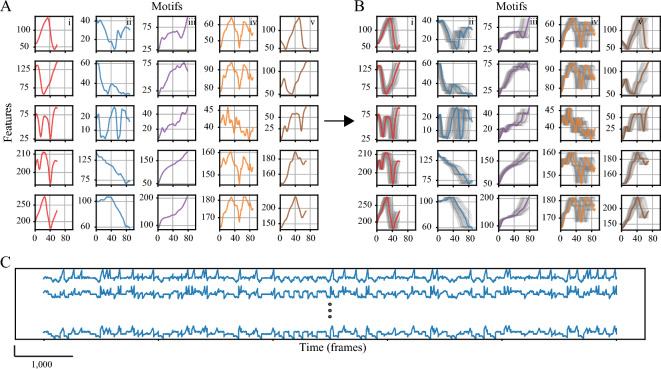
Semi-synthetic data. (**A**) Five motifs from the rat experimental data are presented using selected distances between the landmarks. Each column and color indicate a single motif, the rows depict five features (distances) of each motif. (**B**) The motifs’ variants following warping. (**C**) The resulting continuous signal.

We used semi-synthetic data to assess TASC performance compared to a ground truth. This dataset was generated from data recorded during experiments involving freely behaving rats (Methods—Experimental datasets). Five individual motifs of varying lengths (60–90 frames) were manually identified using videos and extracted from the data. The criteria for the motifs’ selection was a single cycle of a semantically distinguishable behavior different from the previously selected ones. Multiple variants of these motifs were generated by warping each with a uniformly distributed random linear function (± 0.1 for ${\tau }_{w}$, 1 ± 0.2 for ${s}_{w}$) (Fig. 3A). A baseline for the continuous signal was defined as the mean value across motifs and the variants were then inserted in random order. Following normalization to a range between 0 and 1, white noise (Normal (µ = 0, σ = 0.01)) was added to the signal (Fig. 3B).

TASC was applied to the dataset using an initial naïve segmentation using 90 frames per window (0.75 s), *F*_*E*_ = principal component analysis (PCA, 10 components) and *F*_*C*_ = Fuzzy C-Means (FCM, 5 clusters, using median centroids). We chose to use PCA due to its proven reliability and relative simplicity of the dataset. The specific parameters (see Table 2) were set so as to accommodate the relative simplicity of the dataset. For example, $\gamma$ for the outlier removal is set so as to preserve all of the clusters’ members as the initial clustering performed well. We set M = [− 10, 10] and L(n) = [ − n, 10], where n is the running epoch. Lastly, $\alpha ={10}^{R(n)}$ where R is a range of evenly spaced numbers between [0.5, − 1] for the number of epochs. We chose to use dynamic $\alpha$ as with each epoch the overall segmentation should stabilize, and we can permit for stronger warping to fine-tune it.

**Table 2:** TASC parameters for the datasets.

Dataset	Values
“Semi-synthetic”	$M = ( - 10,10),\;{\text{L}}({\text{n}}) = ( - {\text{n}},10),\;\upalpha = 10^{R(n)} \;(R = [0.5, - 1]),\;\gamma = 4$
MotionMapper	$M = \left( { - 10, 10} \right),\;{\text{L}} = \left( { - 5, 6} \right),\;\upalpha = 0.5,\;\gamma = 3.5$
Keypoint-MoSeq	$M = \left( { - 5,5} \right),\;{\text{L}} = \left( { - 1,10} \right),\;\upalpha = 0.5,\;\gamma = 3$
“Rats”	$M = \left( { - 5,5} \right),\;{\text{L}}\left( {\text{n}} \right) = \left( { - {\text{n}},10 - {\text{n}}} \right),\;\upalpha = \left[ {1.2,1.0,0.8,0.6} \right],\gamma = 2$
“Non-human”	$M = \left( { - 2,3} \right),\;{\text{L}}\left( {\text{n}} \right) = \left( { - {\text{n}},2} \right),\;\upalpha = 0.1,\;\gamma = 3$
“Human”	$M = \left( { - 1,2} \right),\;{\text{L}} = \left( { - 1,2} \right),\upalpha = \left[ {0.5,0.3,0.3} \right],\gamma = 3$

**Figure 4:**
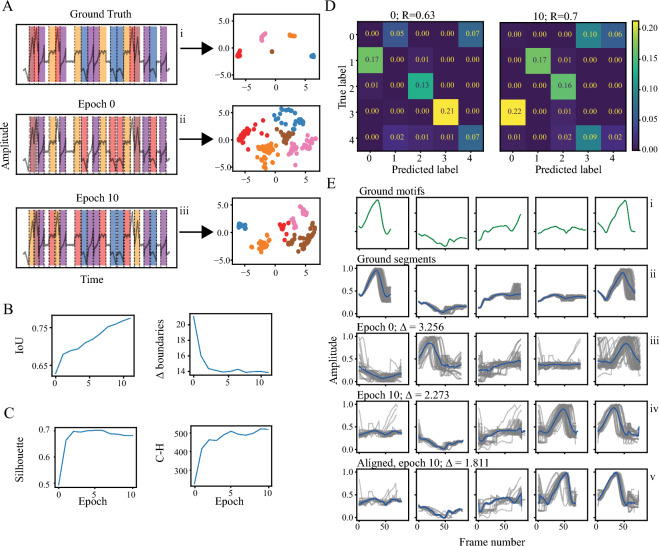
Semi-synthetic data results. (**A**) A part of the signal overlaid with: (i) the ground truth, (ii) the initial segmentation and (iii) following epoch 10 of the TASC segmentation (left); and the corresponding latent space (right). (**B**) Segmentation evaluation metrics applied to the discovered boundaries, IoU (left) and cumulative L1 distance between corresponding boundaries (right). (**C**) Clustering evaluation metrics applied to the found clusters in the embedded space, Silhouette score (left) and Calinski-Harabarz (right) indexes. (**D**) Confusion matrices before and after applying TASC. The matrices are normalized to the overall number of true behaviors. (**E**) (i) The original sub-behaviors, (ii) clusters and their centers given the ground truth boundaries and labels, (iii) initial segmentation and clustering, (iv) segmentation and clustering after TASC, (v) clusters aligned using the found warping parameters. The average weighted distances between cluster members and cluster centers shown on top. Only one feature is presented to improve visual clarity.

The alignment of the segments’ boundaries with the ground truth and segments’ clustering improved over epochs (Fig. 4A). Notably, the clusters’ order is not preserved by TASC, but may be added post hoc using the confusion matrix. The boundary precision enhancement is reflected in the segmentation metrics, such as Intersection over Union (IoU) and the average distance between the corresponding boundaries (Δ boundaries) (Fig. 4B). The clustering evaluation metrics follow the same dynamics (Fig. 4C). TASC capacity to place flexible boundaries and select segments of varying length leads to an increase in the fraction of true segments identified, reflected in the confusion matrices (Fig. 4D). Segmentation and clustering enhancement can also be observed when comparing the segments in the temporal domain (Fig. 4E).

### TASC initiation with existing methods

We conducted a comparative analysis with MotionMapper and Keypoint-MoSeq, the recent extension to MoSeq. We used the semi-synthetic data, to evaluate the results against the ground truth as in the previous section. We note that the latter operates directly on landmarks, and as such we generated the semi-synthetic data for it using the landmarks and not pairwise distances. We then used the distances for TASC as before. We evaluated whether TASC succeeded in enhancing the accuracy of segmentation and clustering.

The parameters for both were empirically fine-tuned so that the results form an adequate basis for both evaluation and uncovering the underlying patterns. (1) MotionMapper—The dimensionality reduction is performed with PCA, and 3 components that explain 95% of variance are used further. We chose to compute wavelets with minimal and maximal frequencies being 1 and 4 accordingly with resolution 30 and to use UMAP for data embedding. (2) Keypoint-MoSeq—We set the *kappa* parameter to 1e4. We found that this parameter produced segmentation comparable to the ground truth. As Keypoint-MoSeq does not receive a parameter tied to the number of clusters, it produced many clusters, some of which with very few members. However, as expected, most segments fell within 5 clusters. We retained only those 5 clusters with the highest population following an educated guess that they would represent the underlying patterns.

**Figure 5:**
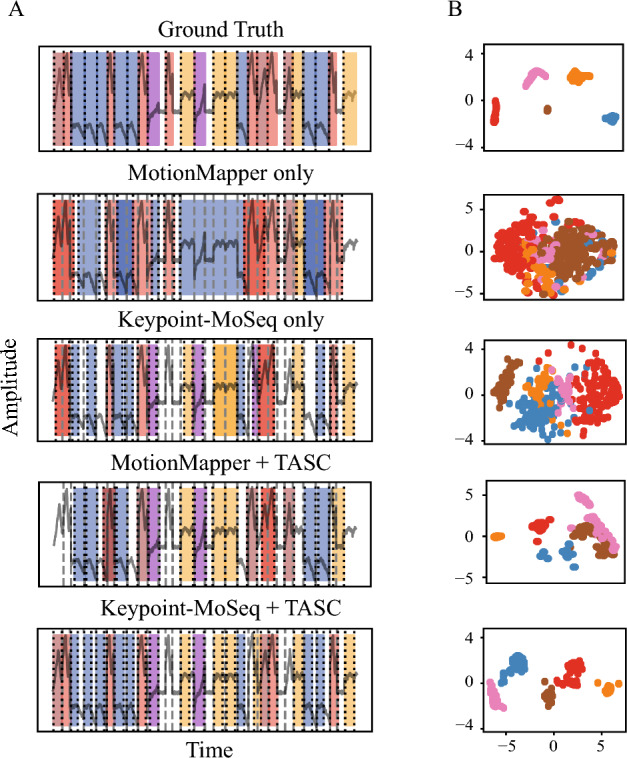
TASC improvement of MotionMapper and Keypoint-MoSeq initial segmentation. (**A**) A part of the signal (1 out of 5 features) overlaid with (top to bottom): the ground truth, results of MotionMapper and Keypoint-MoSeq, results of TASC initiated with MotionMapper (epoch 10) and Keypoint-MoSeq (epoch 5). (**B**) The corresponding latent spaces.

Evaluation was performed using the same metrics as the ones used in the previous section. For fairness and comparative results, we utilized the activity periods calculation and adjusted the results to trim the “rest” from the segments’ tails. We then initiated TASC with the frameworks’ results. Details on parameters for this and subsequent sections can be found in Table 2. TASC showed an improvement in the segmentation and clustering (Fig. 5). For MotionMapper, IoU increased from 0.35 to 0.61, and for Keypoint-MoSeq—from 0.48 to 0.69. Similarly, Recall increased from 0.39 and 0.63 to 0.7 and 0.9, accordingly. TASC broke down longer sequences into discrete motifs for MotionMapper and incorporated previously unseen segments for Keypoint-MoSeq. For the latter, we note that those segments may exist in the original segmentation but may have been discarded either as belonging to a minor class or during “rest” trimming. While in both scenarios, the average distance within clusters decreased, we noticed that when initiated with a sufficiently good existing solution, TASC suffered from propagating some of the pre-existing centroids. This may lead to less clearly defined centroids that do not best reflect the underlying motifs. We elaborate this issue further in Discussion.

### TASC application to experimental data

#### Rat data

**Figure 6:**
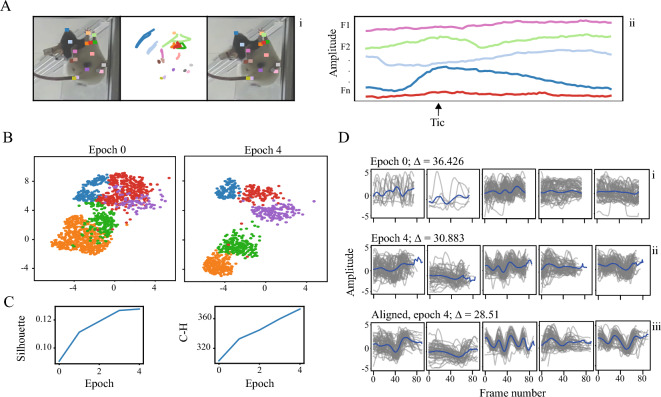
Rat data. (**A**) Example of a movement motif from a freely behaving rat. The motif is shown as either: (i) the first (left) and last (right) frame of the movement and the landmark tracking in 2D space between them (center) and (ii) a subset of the features over time. (**B**) A sample of five clusters formed in the data in latent space before and after TASC (**C**) Evaluation metrics for the clustering. (**D**) The sample clusters in the temporal domain: (i) initial segmentation and clustering, (ii) segmentation and clustering after TASC, (iii) clusters aligned using the identified warping parameters. The average weighted distances between cluster members and cluster centers shown on top. Only one feature is presented to improve visual clarity.

We applied TASC to a dataset of the freely behaving rats (Methods—Experimental datasets) (Fig. 6A). We computed pairwise *distances* between the 13 tracked landmarks and used a pretrained variational autoencoder (VAE) as our embedding function ${F}_{E}$; trained on a window of 75 frames^29^ (input dimension 75*136, output dimension 20). We chose VAE over the standard PCA due to the high complexity of the dataset and the behaviors embedded in it, and window of 75 empirically as the average time scale of the observed motifs. We performed spatiotemporal dilution, which reduced temporal dependency for the closely situated latent vectors, to remove unnecessary overlaps from the initial segmentation, particularly given the high video acquisition rate. Following this, we clustered the data into 25 clusters using FCM. The number of clusters was decided with video grids and evaluation of clusters’ differentiation. TASC showed the following improvements: the embedded space exhibited a progression towards distinctly separated clusters, which is supported by the clustering metrics (Fig. 6B,C). The clusters displayed a progressive alignment in the temporal domain, similar to the observations on semi-synthetic data (Fig. 6D).

#### Non-human primate data

**Figure 7:**
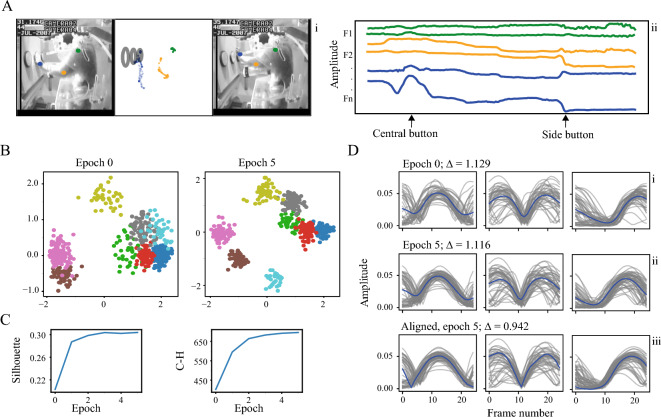
NHP data. (**A**) Example of a movement motif from a non-human primate. (**B**) A sample of three clusters formed in the data in latent space before and after TASC (**C**) Evaluation metrics for the clustering. (**D**) The sample clusters in the temporal domain: (i) initial segmentation and clustering, (ii) segmentation and clustering after TASC, (iii) clusters aligned using the found warping parameters. The average weighted distances between cluster members and cluster centers shown on top. Only one feature is presented to improve visual clarity.

We applied TASC to a dataset acquired from NHPs performing a simple sensorimotor association task (Methods—Experimental datasets) (Fig. 7A). We used the positions of the elbow and palm markers of a NHP following their normalization to a range between 0 and 1, these two joints’ velocity and an angle between all three joints, with the elbow being the vertex. The subsequent process is identical to the one described in the previous sections. For the initialization, we used window size of 25 frames based on visual assessment of the motifs of interest, PCA with number of components explaining 85% of variance and 45 clusters, number of which was predicated by the Silhouette method and visual assessment of the clusters within the videos. TASC showed improved clusters separation, as evident in the latent space (Fig. 7B,C) and better-defined movement motifs represented by clusters’ centers (Fig. 7D).

#### Human data

**Figure 8:**
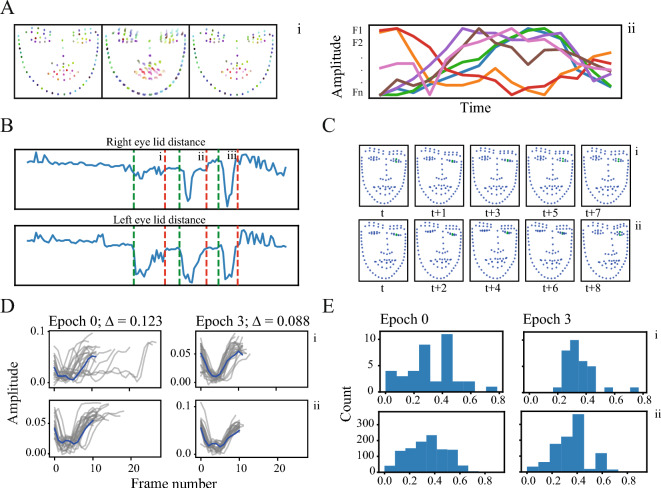
Human patients’ data. (**A**) Example of a movement motif from a Tourette syndrome patient. (**B**) A segment of the signal: original distances (blue), low pass filtered version (orange). The vertical lines mark two tics: (i) left eye tic and (ii) tic involving both eyes; and (iii) a regular blink. Green marks the landmarks, distance within which is described in the following subfigures (left eyelid). (**C**) The tics depicted using the landmarks. (**D**) Temporal distribution of the two clusters of tics before (left) and after (right) TASC. Only one feature is presented to improve visual clarity. (E) Distribution of the timing of the minimum distance (closing of the eye) relative to the segment’s length before (left) and after (right) TASC for the two tics.

Finally, we used TASC on a dataset recorded from Tourette syndrome patients whose faces were recorded while being engaged in multiple tasks (Methods—Experimental datasets) (Fig. 8A). In this task our aim was to assess whether TASC can successfully align an existing labeling. We preprocessed the data similarly to the previous sections while applying a low-passed filter due to a high level of noise in landmark identification. This dataset contained manual annotations of the tic timing and type, which could be used for assessment of the movement segmentation and clustering, respectively. As a result, we omitted ${F}_{C}$ and *F*_*E*_ altogether and used IoU scores with the ground truth for segments’ labeling instead. We chose the tics with the highest occurrence rate as our clusters, specifically a tic involving one eye (Fig. 8Bi,Ci) and a tic involving two eyes (Fig. 8Bii,Cii). The timing of the manually labeled tics was highly variable in both phase and duration (Fig. 8D left). TASC improved the tic alignment as evident in the synchronization of the tics’ peak (Fig. 8D right), characterized by the timing of the minimum distance between the eyelids relative to the segment (Fig. 8E).

## Discussion

TASC is a framework for segmentation and clustering of behavioral data which may be applied in conjunction with existing pipelines that use dimensionality reduction and clustering. It receives as input a multidimensional signal and a method for segments’ dimensionality reduction and cluster assignment, and iteratively refines segmentation boundaries and cluster assignments through linear alignment applied to segments of varying length, followed by re-embedding and re-clustering until convergence is achieved. Linear alignment maps the timing of time series sequences, allowing a more accurate comparison for sequences that share an underlying shape while minimizing the effect of the temporal dissimilarity. We demonstrated the enhanced segmentation and clustering initially on a semi-synthetic dataset based on an experimental rat dataset, which enabled the assessment of the framework performance relative to a well-defined ground truth. This demonstrated, (1) an improvement in the number of detected and correctly labeled segment, reflected in increased IoU between the ground truth and TASC’s segmentation, (2) improved boundary accuracy reflected in decrease of average L1 distance between segmentation and the ground truth counterparts’ boundaries, and (3) distinct clusters with well-defined underlying motifs as seen in the temporal and latent spaces. We then showed TASC’s applicability to enhancing an existing pipeline by initiating the framework with MotionMapper and Keypoint-MoSeq. Consequently, TASC was applied to three experimental and clinical datasets, displaying its wide range of application. Although these datasets vary significantly, TASC demonstrated consistent enhanced results as evident through advancements in the embedded space, mean distance of clusters’ members to their respective centroids, and centroids’ shape definition. As the main principle behind the design is generalizability, we do not enforce any specific choice of the input functions, *F*_*E*_ and* F*_*C*_. Moreover, depending on the task and data, there may be no need or benefit for using *F*_*E*_ or even both of the functions. For the former, in principle it is possible to use DBA^25^ or a similar method to directly cluster time series. The latter was demonstrated in our experiment on the “Human” dataset, where we did not employ neither embedding nor clustering function.

Behavior motifs exhibit spatiotemporal variability, specifically repetitions of an individual sub-behavior can have different temporal profile. Behavior segmentation and clustering should be robust to this issue, i.e., assign similar movement performed at different speeds and captured at different stages into the same group, accommodating variations in repetitions’ duration and phase. Failure to achieve this robustness results in inaccurate segmentation and misleading clustering that does not reflect the underlying behavior. Adding a warping parameter to account for behaviors' temporal variability was shown to combat over-segmentation and improves overall results^30^. We address the temporal variability by calculating warping parameters using linear alignment for continuous segments. Our hypothesis is that addressing the variability using such a metric leads to a more accurately described space, where the neighboring members are more likely to represent the same underlying behavior.

We couple segmentation and clustering in an iterative manner. We first refine the existing clusters by comparing segments to their clusters’ centroids, each time selecting the ones that are not only closer to the centroid after alignment, but also require the least warping for the said alignment. This effectively leads to re-segmentation supported by the enhanced clustering. We then iterate over the process to achieve the best clusters and their centroids, as each iteration only the highest scoring candidates are selected, leading to clusters alignment to a single phase. Additionally, these warping parameters account for each member’s temporal variability and are utilized for further enhancement of the embedded space and clustering as a result.

Current behavior analysis solutions such as MotionMapper^19^, B-Soid^14^ and MoSeq^13^, may perform unevenly on different datasets depending on the experimental subject, setup and even recording sensors. For example, MotionMapper, which uses spectral data characteristics, is most suitable for subjects generating clearly cyclic behavior, such as fruit flies, while MoSeq was designed for behavior analysis using depth cameras and targeting mice. Keypoint-MoSeq^20^ generalizes to the case of keypoints extracted using non-depth cameras, however it has not been evaluated on non-rodents to the best of our knowledge. In regard to human clinical data, there exists a plethora of solutions in HAS that gained interest both in academia and industry^31–35^. Zhou et al. used DTW kernel to enhance segmentation and achieve temporal invariance^26^. The framework is designed to process the entire data stream at once and given the inherent computational costs they provide a method for downsampling based on redundant frames. While temporal reduction was shown to reliably down-sample normal human movement data, it does not fit to the abrupt and rapid movement often present in clinical population. Additionally, being non-linear, DTW is not easily interpretable or transferable to other domains, such as neural recordings^9^. Zhang et al. proposed a two-step solution, where firstly, Spectral Clustering (SC) is employed to learn initial patterns and their associated statistics, and secondly these statistics are used to cluster and then segment new input^36^. This approach yielded impressive results on human action tasks such as TUM Kitchen^37^, but such datasets deal with multi-second time scales rather than the time scale of the data we describe. Overall, depending on the research aims, its subjects and the need for precision, one may choose and apply any of the abovementioned solutions accordingly and the results may suffice. However, if further boundary precision and movement alignment is required, as may be the case when the data is coupled with neuronal recordings, we propose a refinement of these solutions using TASC.

An important property of TASC is the parameter estimation for the temporal warping and phase alignment of the segments within each cluster. This enables an extension to other domains of the research using these parameters. They may be used to analyze behavior and its characteristics under different conditions^30^. Moreover, they may be used in the domain of neuronal recordings: in a recent work, Williams et al. shows that linear alignment can be effectively used on repetitive trials to align neural data to discover otherwise hidden meaningful patterns and analyze them^9^. Their framework works independently from behavior analysis and addressed trial organized experiments. TASC and its alignment parameters enable an extension of this method to study unconstrained behavior, which does not necessarily reside within a repeating trial-based structure. The parameters derived from behavior may be applied to the corresponding neuronal recordings to test whether the firing patterns are similarly misaligned.

As TASC was designed within the paradigm that behavior may be viewed as a sequence of discrete motifs, it is best fit for tasks where such repetitive motifs are expected and are the focus of the research. TASC is hence less suitable for tasks such as HAS, where longer periods of activity are of interest (for example full episodes of multi-second walking, jogging or food cutting). In such cases, TASC output for identifying the short motifs may be hierarchically accumulated and used to define larger scale bouts of activity. In the clinical setting, TASC may be used for refining manually marked segmentation and clustering. Visual assessment of behavior by trained experts remains the most robust way to confirm the number of clusters, their quality and temporal boundaries. However, even this assessment is prone to human factor mistakes and subjectivity. There may be inconsistencies between the trained experts, within sessions or even within the sub-behaviors performed in different contexts of a session^38^. The TASC framework may be applied to alleviate the problem as was shown in the Tourette’s patients' section, in which improved alignment of the eye tics reflected in the tics’ climax synchronization is achieved. Alternatively, the linear alignment scores may be used for flagging segments with a high distance for reconsideration.

There are certain limitations to TASC, including its reliance on clear structural contributions of the motifs to the input signal. A poor choice of features may lead to centroids with little or no meaningful variation. Distance and alignment to such centroids will not significantly differ for members of different clusters. As such, the framework could benefit from semi-supervision in the form of ground truth motif patterns provided as initial centroids. Experimenters tend to perform exploratory data analysis (EDA) before turning to rigorous data analysis, which means some motifs in the data could be known and manually assessed to be used as an input to the framework. Alternatively, MP could be used to make an educated guess on the existing top-k motifs in the data^18^, i.e., potential centroids. Another limitation is that TASC results are not simply transferable across sessions. However, it does reveal aligned patterns of behavior which may be similarly used for improved initialization and hence smaller number of TASC iterations on novel data. Lastly, as mentioned previously (Methods—Weighted Euclidean distance), the current metric is not amplitude-invariant and depending on the research needs, we suggest substituting it with a correlation-based metric instead.

The TASC framework uses linear alignment for its simplicity and explainability. It is expandable to piecewise linear alignment, which allows higher flexibility while remaining explainable and transferable. Piecewise alignment optimizes multiple warping parameters for sub-segments of signal, as opposed to one parameter, i.e., such alignment could better fit behaviors that can vary non-uniformly over the course of the action. Extension to DTW may also be performed allowing even further flexibility at the cost of reduced explainability and transferability. This range of solutions may be used depending on behavior complexity.

The high variability of behavioral data in both human subjects and experimental animal models has led to a wide range of behavior quantification tools. TASC aims to complement those tools with a generalized framework which will increase their usability, precision, and robustness. We believe this notion of generic refinement of segmentation and clustering is applicable to time series data arising from a wide range of fields and that TASC may become a basis of inspiration for future such works.

## Methods

TASC constitutes a generalized customizable framework that addresses a wide variety of experimental scenarios. In addition, the framework supports a complete processing pipeline providing basic algorithms for the processing stages that do not constitute the core algorithmic part of the framework.

### Initial segmentation and clustering

While TASC accepts any external initial segmentation and clustering of the data, it also contains the internal simplified method for initialization. Overlapping segments are naively cut to a given initial window size. The segments are dimensionally reduced using any embedding function (*F*_*E*_), which is PCA by default. For clustering of the embedded space, any clustering function (*F*_*C*_) may be used, with Fuzzy C-Means (FCM) by default^39,40^. Using FCM utilizes the soft labels to select the best candidates while eliminating any overlaps through dynamic programming (see section below). Alternatively, the distance measure of any F_C_ can be transformed into scores to be used for that process. The number of clusters is selected based on the Silhouette method^41^, supported by visual assessments involving plotting the data across clusters. In cases where videos are present, video grids may be used to observe the behavior context of the clusters. The resulting segmentation and clustering obtained through this process then serve as the initial input to TASC, if an alternative is required for an external input.

### Segment selection and overlap elimination

TASC optimizes segmentation score while enforcing no segment overlaps. To achieve this, given potentially multiple overlapping candidates, we use dynamic programming (DP) (Box 2). The objective of the DP is to maximize the cumulative score of the selected segments while allowing no overlaps. For fast lookup, we use Interval Tree (https://pypi.org/project/intervaltree/). The DP assumes segments’ scores as input, however, in situations where costs are provided, it is possible to convert them into scores. For this, we normalize the costs on a per-cluster basis, to avoid values skewness in imbalanced clusters, and deduct them from the total maximum value of 1.

Box 2. Pseudocode for overlap elimination **Definitions:**

Number of segments     *N*

Scores     $$S=\{{s}_{1},{s}_{2},\dots {s}_{N}\}$$*,*

Boundaries     $${T}_{i}=\left({t}_{B}, {t}_{E}\right), {t}_{{B}_{i}}<{t}_{{B}_{i+1}}, 1\le i\le N$$

Accumulated score     * K*_N*N_

Index mapping     *I*_N*N_

**Algorithm:**

$$K\left[0,:\right]\leftarrow S$$

$${K[i, :]\leftarrow -\infty }, 1\le i\le N$$

for $$i=1, N$$:

  for $$j= 0, N$$:

    if *T[i-1]* and *T[j]* do not overlap:

$$I\left[i-1,j\right]\leftarrow [I\left[j\right], argmax\left(K\left[j+1\right]\right), i-1]$$

$$K\left[i,j\right]\leftarrow K[0,i-1]+max(K\left[j+1\right])$$

Backtrack path *p* based on the map $I$ and return* p*

### Outlier removal

Removal of outlier cluster members improves the centroids’ definition and subsequent clustering results. We compute pairwise distances between members of each cluster using the weighted Euclidean distance (see section below). Members that exceed a distance threshold of $\gamma$ standard deviations from the average distance within the cluster are removed. This process may produce gaps in segmentation, which we address by evaluating and reassigning such gaps later (see section below).

### Gap reassignment

Gaps—segments in the signal that are not assigned to clusters—can form due to two primary reasons: (1) outlier removal at the beginning of each epoch and (2) overlap elimination at the end of each epoch. We address these gaps by identifying and reintroducing candidate segments within significantly large gaps. A gap is considered significantly large if its duration is not less than the average duration of the segments in the current iteration. We use overlapping windows to search for the best segments within such gaps. For each window, we compute its distance to all clusters’ centers using Weighted Euclidean distance. If the minimum distance is less than the corresponding cluster’s average distance determined during the outlier removal stage, we reassign the segment. To avoid potential overlaps, we convert the calculated distances into scores and use them to select the best segments and eliminate any overlaps.

### Weighted Euclidean distance

Euclidean distance is the most popular choice when calculating distances between samples, however, it is limited to sequences of the same length. We customized Euclidean distance to handle varying lengths. Given two sequences $\left(x,y\right)$, we first calculate variables the minimum and maximum of their lengths $\left(minlen \text{ and } maxlen \text{ respectively}\right)$. We pad the shorter sequence with this sequence’ median + $\epsilon$ to the *maxlen*; we denote the resulting sequences $(a, b)$ respectively. A custom Hamming window $H$ is used to emphasize the distance in the temporal center of the motifs, as the segment edges tend to differ. To negate preference for shorter sequences, which would be padded more heavily with constant values, we establish the baseline for $H$ as 1 and add the Hamming window of length $minlen$ to it.

$$weightedL2\left(x, y\right)= L2\left(a, b\right)*H$$

$$H=\left[1+hamming\left(minlen\right), {1}_{0},\dots {1}_{maxlen-minlen}\right]$$

The Weighted Euclidean distance modification aims to emphasize shape similarity between sequences of varying lengths. However, it does not accommodate magnitude variability, e.g., moving an arm up partially or entirely. For this, it may be prudent to substitute the modification of the Euclidean distance with a weighted correlation-based measure^42^.

### Linear alignment

For a pair of sequences, we will consider one as a template and the other as a query. The algorithm then searches for a set of parameters $[{\tau }_{w}, {s}_{w}]$*,* that define offset and warping for matching the sequence to the template, such that the distance between the transformed query and the pattern is minimal. As such, it is a minimization problem. Semantically, ${\tau }_{w}$—the offset of the function, and ${s}_{w}$—the slope of the function, represent a shift of the sequence to the past or future and a “shrinking” or “stretching” of the sequence, respectively.

$$\widehat{x}\left(t\right)= interpolate\left(x\left({\tau }_{w}+t*{s}_{w}\right), x\left(({\tau }_{w}+t*{s}_{w})+1\right)\right), t\in [0, 1)$$

For parameter search, we use simplicial homology global optimization (SHGO) to minimize the loss^43^, defined by the sum of the L2 distance between the warped sequence and the template. When unconstrained, it can encounter extreme parameters, especially when the sequences do not share an underlying shape. For this we incorporate a penalty—a weighted regularization term *r*. Setting the weighting parameter $\alpha$ to 0 effectively neglects the penalty.

$$loss=weightedL2\left(\widehat{x}, y\right)+\alpha*r$$

We implement a larger penalty for the warping parameter.

$$r=\mathit{arctan}\left(abs({\tau }_{w}\right))+1.5*\mathit{arctan}\left(abs\left({s}_{w}-1\right)\right)$$

### Experimental datasets

We applied TASC to three experimental datasets that inspired the framework and are actively researched in the lab, and to one semi-synthetic dataset enabling evaluation against the ground truth. The datasets share a common trait of expected motif repetitions. The “Rat dataset” consists of a single GoPro camera (120 frames/s) recording of freely behaving rats (for surgical and experimental details, see^44^). From these recordings,17 landmarks per frame were extracted using a custom stacked-hourglass network^45,46^. The “Non-Human (NHP) Primates dataset” consists of recordings of NHP during a session that includes a simple sensorimotor task but is not limited to it. The upper body was filmed using a side camera with (25 frames/s) during approximately 1 h sessions (for surgical and experimental details, see^47^). We applied DeepLabCut (DLC)^48^ to track three joints of the unconstrained arm: palm, elbow and shoulder. Finally, the “Human dataset” consists of camera recordings of faces of Tourette syndrome patients (Helsinki protocol 0569-19-RMC). They were filmed using the frontal camera of a smartphone at 30 frames/s (for experimental protocol, see Ref.^49^). We used the stacked hourglass network to extract 98 facial landmarks and calculated the landmark distance matrix for each frame^50^. Trained experts, who marked the type and timing of the individual tics, annotated the patient data.

### Evaluation metrics

We use complementing approaches in evaluating semi-synthetic, experimental and clinical data. The semi-synthetic data has an existing ground truth segmentation and clustering, which serves as a reference to demonstrate the trends across different metrics. For the semi-synthetic data, we assessed both segmentation and clustering; while for the experimental data—only the clustering. For the human clinical data, we demonstrate the predefined clusters’ alignment and explore the semantic meaning associated with it. We consider a pair of segments in the ground truth and those produced by TASC as corresponding if their IoU score is at least half of the ground segment’s duration. For the segmentation evaluation, we calculated the average IoU and the sum of L1 distances between the corresponding segment boundaries and the ground truth.

### Cited code

We thank authors for making their work accessible which enabled the comparison:

MotionMapper Python implementation—https://github.com/gordonberman/MotionMapper.git Keypoint-MoSeq—https://github.com/dattalab/keypoint-moseq.git.

## Data Availability

The semi-synthetic dataset used during the current study is available at the mentioned github. The ‘Rats’ and ‘Non-human’ data is available at: 10.6084/m9.figshare.25540291.v1.

## Code availability

The code for TASC is available as a github repository at: https://github.com/ibglab/TASC.
